# Actinobacterial Diversity in Volcanic Caves and Associated Geomicrobiological Interactions

**DOI:** 10.3389/fmicb.2015.01342

**Published:** 2015-12-09

**Authors:** Cristina Riquelme, Jennifer J. Marshall Hathaway, Maria de L. N. Enes Dapkevicius, Ana Z. Miller, Ara Kooser, Diana E. Northup, Valme Jurado, Octavio Fernandez, Cesareo Saiz-Jimenez, Naowarat Cheeptham

**Affiliations:** ^1^Food Science and Health Group (CITA-A), Departamento de Ciências Agrárias, Universidade dos AçoresAngra do Heroísmo, Portugal; ^2^Department of Biology, University of New MexicoAlbuquerque, NM, USA; ^3^Instituto de Recursos Naturales y Agrobiología, Consejo Superior de Investigaciones CientíficasSevilla, Spain; ^4^Grupo de Espeleología Tebexcorade-La PalmaCanary Islands, Spain; ^5^Department of Biological Sciences, Faculty of Science, Thompson Rivers UniversityKamloops, BC, Canada

**Keywords:** Actinobacteria, volcanic lava caves, microbe-mineral interactions, microbial diversity

## Abstract

Volcanic caves are filled with colorful microbial mats on the walls and ceilings. These volcanic caves are found worldwide, and studies are finding vast bacteria diversity within these caves. One group of bacteria that can be abundant in volcanic caves, as well as other caves, is Actinobacteria. As Actinobacteria are valued for their ability to produce a variety of secondary metabolites, rare and novel Actinobacteria are being sought in underexplored environments. The abundance of novel Actinobacteria in volcanic caves makes this environment an excellent location to study these bacteria. Scanning electron microscopy (SEM) from several volcanic caves worldwide revealed diversity in the morphologies present. Spores, coccoid, and filamentous cells, many with hair-like or knobby extensions, were some of the microbial structures observed within the microbial mat samples. In addition, the SEM study pointed out that these features figure prominently in both constructive and destructive mineral processes. To further investigate this diversity, we conducted both Sanger sequencing and 454 pyrosequencing of the Actinobacteria in volcanic caves from four locations, two islands in the Azores, Portugal, and Hawai'i and New Mexico, USA. This comparison represents one of the largest sequencing efforts of Actinobacteria in volcanic caves to date. The diversity was shown to be dominated by *Actinomycetales*, but also included several newly described orders, such as *Euzebyales*, and *Gaiellales*. Sixty-two percent of the clones from the four locations shared less than 97% similarity to known sequences, and nearly 71% of the clones were singletons, supporting the commonly held belief that volcanic caves are an untapped resource for novel and rare Actinobacteria. The amplicon libraries depicted a wider view of the microbial diversity in Azorean volcanic caves revealing three additional orders, *Rubrobacterales, Solirubrobacterales*, and *Coriobacteriales*. Studies of microbial ecology in volcanic caves are still very limited. To rectify this deficiency, the results from our study help fill in the gaps in our knowledge of actinobacterial diversity and their potential roles in the volcanic cave ecosystems.

## Introduction

Actinobacteria are an ubiquitous phyla found to thrive in almost any environment, from soil and marine, to less expected environments such as insects, plants, roots, and caves (See Tiwari and Gupta, 2013; Subramani and Aalbersberg, 2013 for reviews). Recent culture independent studies have found Actinobacteria in high abundance in a variety of cave types, including volcanic caves (Pašić et al., 2010; Northup et al., 2011; Cuezva et al., 2012; Niyomyong et al., 2012; Quintana et al., 2013; Barton et al., 2014; Hathaway et al., 2014). Furthermore, many characterized species of Actinobacteria have been described from caves (Groth et al., 1999; Lee et al., 2000, 2001; Jurado et al., 2005a,b; Lee, 2006).

Primary and secondary metabolites from Actinobacteria have been described as important sources of industrial compounds (Miao and Davies, 2010). Rare Actinobacteria, important for novel secondary metabolite production, have been found in many different soil types (Tiwari and Gupta, 2012; Guo et al., 2015), but caves, volcanic caves included, remain an underexploited environment to screen for industrially important compounds. Goodfellow and Fiedler (2010) suggested examining underexploited sources of Actinobacteria and using taxonomic diversity as a surrogate for chemical diversity, based on the assumption that novel species may contain unique compounds, reducing the re-discovery of the same handful of known secondary metabolites.

Cave Actinobacteria are of particular interest because of the unique environment in which they live. The extreme (i.e., low nutrient inputs, low productivity) and often pristine environment would result in bacteria exploiting different metabolic pathways, including the capacity for biomineralization and rock-weathering (Cuezva et al., 2012; Miller et al., 2012a,b). Caves are characterized by microenvironments, which result from several types of reactions, including microbial processes that often involve redox reactions (Barton and Northup, 2007). These mineral microniches control the diversity of subsurface microbial populations (Jones and Bennett, 2014), since microbial colonization of rock surfaces is driven by the rock's chemistry and the organism's metabolic requirements and tolerances, suggesting that subsurface microbial communities have specific associations to specific minerals. In fact, caves on Earth can harbor a wide variety of mineral-utilizing microorganisms that figure prominently in the formation of secondary mineral deposits and unusual mineralized microstructures recognized as biosignatures. Tubular mineralized sheaths (Boston et al., 2001; Northup et al., 2011), bacteria concealed within mineral deposits (Northup et al., 2011), microfossils preserved in minerals (Provencio and Polyak, 2010; Souza-Egipsy et al., 2010), filamentous fabrics (Hofmann et al., 2008) and “cell-sized” etch pits or microborings produced by bacteria (McLoughlin et al., 2007) are some of the proposed models for biosignatures found in subsurface environments.

The main goal of the research presented here is to obtain a better understanding of the actinobacterial diversity in volcanic caves from different parts of the world. Comprehensive studies on microbial community ecology of caves identifying abundant, rare and novel species and their environmental implications are still scarce. In the course of this study, we aim to unravel the diversity and composition of volcanic cave Actinobacteria, some of the biogeochemical role of Actinobacteria in caves and their geomicrobiological interactions. Recently, a rapid expansion of interest in subsurface environments has emerged to better understand biodiversity, origins of life on Earth and on other planets. In fact, the reported early results on liquid water and rather recent volcanic activity yielding volcanic caves on Mars, suggesting that the Martian subsurface can house organic molecules or traces of microbial life (Léveillé and Datta, 2010; Northup et al., 2011), make the search for microbial life on Earth's volcanic caves even more compelling. Overall, this work helps us to understand whether volcanic caves under study present similar levels of diversity and do Actinobacteria found in volcanic caves show diversity across different scales from community level to morphology to microbe-mineral interactions.

## Materials and methods

### Morphological characterization of colored microbial mats

#### Sampling of Azorean, Canadian, Canarian, Hawaiian, and New Mexican Volcanic Caves

**Figure 1:**
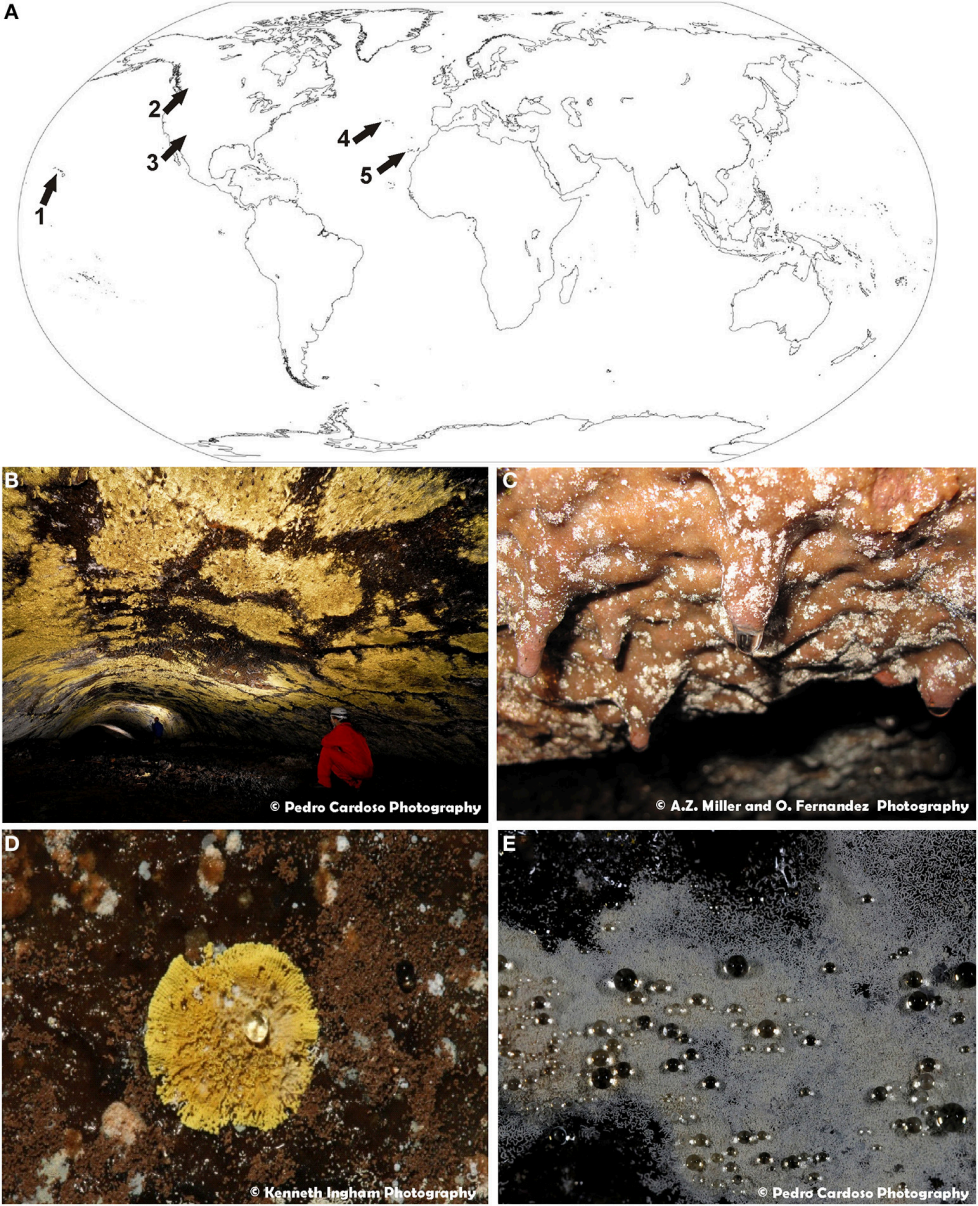
**(A)** World map of volcanic caves studied in this work: 1—Hawaiian volcanic caves (U.S.A.); 2—Helmcken Falls Cave, British Columbia (Canada); 3—New Mexico volcanic caves (U.S.A.); 4—Azorean lava caves (Portugal); 5—La Palma caves, Canary Islands (Spain). **(B)** General view of extensive yellow microbial mats from Gruta da Terra Mole (Azores, Portugal). **(C)** General view of white colonies forming dendritic branches on basaltic lava from Fuente de la Canaria cave (La Palma Island, Spain). **(D)** Close-up view of a yellow colony from Gruta dos Montanheiros (Azores, Portugal). **(E)** Close-up view of white microbial mat covered with water droplets from Gruta da Terra Mole (Azores, Portugal).

Samples of visible white and/or yellow microbial mats on volcanic cave walls and ceilings (Figure 1) were collected from: (1) Bird Park Cave and Kipuka Kanohina Cave System, Hawai'i (USA); (2) Helmcken Falls Cave, British Columbia (Canada); (3) Cave 12 from El Malpais National Monument, New Mexico (USA); (4) Gruta de Terra Mole and Gruta dos Montanheiros in Terceira and Pico Islands, Azores (Portugal), and (5) Fuente de la Canaria, Falda de La Horqueta, Llano de los Caños and Honda del Bejenado caves in La Palma Island, Canary islands (Spain). Samples were taken by gently scraping the colored microbial mats with a sterile scalpel, gathering it into sterile vials and stored at 4°C until laboratory procedures.

#### Scanning electron microscopy

Bulk samples with microbial mats from Canarian volcanic caves (Spain) were directly mounted on a sample stub and sputter coated with a thin gold/palladium film. Samples were subsequently examined on a Jeol JSM-7001F field emission scanning electron microscope (FESEM) equipped with an Oxford X-ray energy dispersive spectroscopy (EDS) detector. FESEM examinations were operated in secondary electron (SE) detection mode with an acceleration potential of 15 kV at Instituto Superior Tecnico, University of Lisbon, Portugal. Samples from Helmcken Falls Cave (Canada) were prepared, processed, and observed at the University of British Columbia (UBC) BioImaging Facility (Cheeptham et al., 2013). Rock chips with microbial mats from Azores, New Mexico, and Hawai'i were mounted, processed and observed as described in Hathaway et al. (2014).

### Estimation, description, and novelty of actinobacterial diversity

#### Sample collection and clone library preparation and OTU-based analysis for New Mexico (USA), Hawai'i (USA), and Azores Islands (Portugal)

Microbial mat samples of various colors were collected from the dark zone of five caves (Cave 12, Cave 255, Cave 266, Cave 261, and Cave 315) from El Malpais National Monument, New Mexico, six caves on the Big Island of Hawai'i (Bird Park, Epperson's, Kaumana, and Thurston Caves and the Maelstrom and Kula Kai Caverns Sections of the Kipuka Kanohina Cave System), four caves on the Azorean island of Pico (Furna do Lemos, Gruta dos Montanheiros, Gruta da Ribeira do Fundo, and Gruta das Torres) and 11 caves on the Azorean island of Terceira (Algar do Carvão, Gruta das Agulhas, Gruta da Achada, Gruta dos Buracos, Gruta dos Balcões, Gruta da Branca Opala, Gruta da Madre de Deus, Gruta do Natal, Gruta da Terra Mole, Gruta dos Principiantes, and Gruta da Malha), see Figure 1 and Supplemental Table 1. DNA from microbial mats of various colors was aseptically collected. DNA was extracted and purified using the MoBio PowerSoil™ DNA Isolation Kit using the manufacturer's protocol (MoBio, Carlsbad, CA), with the exception of the substitution of bead beating for 1.5 min (Biospec Products, Bartlesville, OK, USA) instead of vortexing for cell lysis. 16S rDNA sequences were amplified with universal bacterial primers 46 forward (5′ -GCYTAAYACATGCAAGTCG- 3′) and 1409 reverse (5′ -GTGACGGGCRGTGTGTRCAA- 3′) (Northup et al., 2010).

Amplification reactions were carried out in a 25-μL volume with 1X PCR buffer with 1.5 mM Mg^2+^, 0.4 μM of each primer, 0.25 mM of each dNTPs, 5 μg of 50 mg/mL BSA (Ambion, Austin, TX, USA) and 1U AmpliTaq LD (Applied Biosystems, Foster City, CA, USA), and carried out under the following thermocyling conditions on an Eppendforf Mastercycler 5333 (Eppendorf, Hauppauge, NY, USA): 94°C for 5 min, followed by 31 cycles of 94°C for 30 s, 50°C for 30 s, 72°C for 1.5 min, with a final extension at 72°C for 7 min. Amplicons were cleaned and purified using the Qiagen PCR cleanup kit (Qiagen, Germantown, Maryland) and cloned using the TOPO TA Cloning kit (Invitrogen, Carlsbad, CA). Sequencing was carried out at the Washington University Genome Sequencing Facility. The subset of Actinobacteria were identified with RDP classifier (Maidak et al., 2001), and used for further analysis.

Alignments of the resulting actinobacterial sequences set were generated using INFERNAL (Nawrocki et al., 2009), trimmed to 104–1403 bp to remove ragged ends, and clustered into Operational Taxonomic Units (OTUs) at 97% similarity with QIIME using uclust (Caporaso et al., 2010). Taxonomy was assigned using uclust against the greengenes 13.8 database (Edgar, 2010; McDonald et al., 2012). Sequences were compared with the GenBank database in March 2015 using the Basic Local Alignment Search Tool (BLAST)^1^ to determine closest relatives (Altschul et al., 1997). An identity matrix was generated using Bio Edit^2^. The tree was built using FastTree with the gamma and nt options (Price et al., 2009, 2010). OTUs and location were added to the tree using the phyloseq package in R (McMurdie and Holmes, 2013; R Core Team, 2015).

All other OTU-based approaches were performed with software package mothur 1.34 (Schloss et al., 2009). Rarefaction curves, non-parametric diversity indexes npsShannon (Chao and Shen, 2003), Shannon (Shannon, 1948) and Simpson (Simpson, 1949) and estimator Chao1 (Chao, 1984), as well as the Good's Coverage (Good, 1953) were calculated to infer the richness and evenness of the samples.

#### 16S rRNA gene amplicon library preparation, pyrosequencing, bioinformatics, and OTU-based analysis in Azorean Volcanic Caves

16S rRNA gene amplicon libraries were prepared from the previously described Azorean microbial mat samples collected from the previously mentioned caves with the exception of Algar do Carvão (Supplemental Table 1). The small subunit rRNA gene was amplified from community DNA targeting the V1 and V3 hypervariable region, with barcoded fusion primers containing the Roche-454 A and B Titanium sequencing adapters, a eight-base barcode sequence, the universal forward primer 5′– AGRGTTTGATCMTGGCTCAG -3′ and the universal reverse primer 5′–GTNTTACNGCGGCKGCTG-3′. Amplicon 454 pyrosequencing, as originally described by Dowd et al. (2008), was performed with PCR amplification as described in Brantner et al. (2014). Following PCR, all amplicon products from different samples were mixed in equal concentrations and purified using Agencourt Ampure beads (Agencourt Bioscience Corporation, MA, USA). Samples were sequenced utilizing Roche 454 FLX titanium instruments and reagents and following manufacturer's guidelines.

The raw pyrosequencing reads were processed using version 1.34 of the mothur software package (Schloss et al., 2009). Sequencing reads were assigned to the appropriate samples based on the corresponding barcode and were quality filtered to minimize the effects of random sequencing errors, by eliminating sequence reads < 200 bp, sequences that contained more than one undetermined nucleotide (N) and sequences with a maximum homopolymer length of 8 nucleotides. Identification and removal of chimeras was performed with Chimera.uchime (Schloss et al., 2011). Sequences not passing these quality controls were discarded. When preparing the inputs for analysis, the “remove.groups” command was used to discard all sequences not belonging to the phyla Actinobacteria.

OTUs were assigned from the uncorrected pairwise distances between aligned 16S rRNA gene sequences, using the average neighbor clustering (Schloss and Westcott, 2011), considering a cut-off value of 97% similarity. All OTU-based approaches were performed with software package mothur 1.34 (Schloss et al., 2009) as well as the taxonomic assignment of the sequences, performed by the Greengenes-based alignment using default parameters. A list of GenBank accession numbers is provided in Supplemental Table 2.

## Results and discussions

### Morphology of colored microbial mats and associated microbe-mineral interactions

One of the important factors influencing the microbial diversity of subsurface environments is the mineral microniches they develop on (Jones and Bennett, 2014). In order to broaden our understanding of the interactions of microorganisms in volcanic caves and their diversity around the world, an extensive SEM study was performed. Colored microbial mats with different morphologies from Azorean, Canadian, Canarian, Hawaiian, and New Mexican volcanic caves were investigated (Figure 1A). Abundant white and yellow microbial mats were distinctly visible to the naked eye (Figures 1B,C). These colored mats may consist of large, dense expanses of microorganisms with coarse and irregular edges covering extensive areas of volcanic cave walls and ceilings (Figure 1B) or small colonies spread all over the surface (Figure 1C). Some colonies adopted the form of white spots with irregularly radiate pattern (Figure 1C) or yellow, round and isolated spots with a symmetrical character (Figure 1D). They can grow on the rock surfaces or on secondary mineral deposits, such as ooze-like deposits frequently found in these volcanic caves. In general, the microbial mats have finely granular surface (Figure 1D) and act as water condensation points, being covered with water droplets, particularly during the wet seasons (Figure 1E).

**Figure 2:**
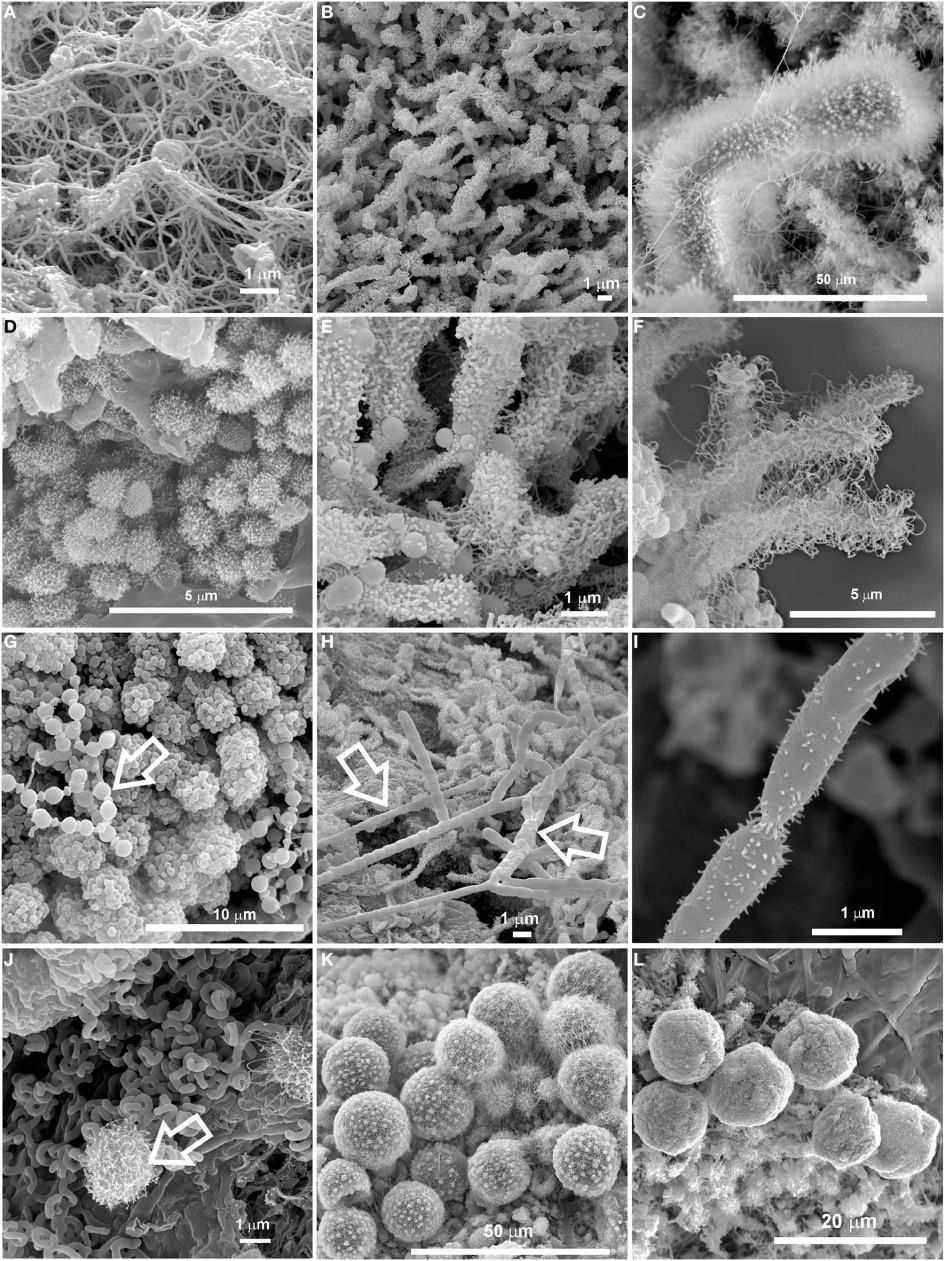
**SEM images of colored microbial mats found in Azorean, Canadian, Canarian, Hawaiian and New Mexican volcanic caves showing a large variety of microbial morphologies and spore surface ornamentation. (A,B)** Dense network of interwoven filaments in Honda del Bejenado and Fuente de la Canaria caves (La Palma Island, Spain); **(C)** Dense masses of *Streptomyces*-like spore chains with hairy ornamentation from Cave 12 in El Malpais National Monument (New Mexico, U.S.A.); **(D)** Coccoid cells with surface appendages or obtuse protuberances from Gruta da Terra Mole (Terceira Island, Azores, Portugal); **(E)** Detailed view from **(B)** showing coccoid cells and clumps of spore chains with obtuse protuberances and surface appendages; **(F)** Close-up view of clusters of *Streptomyces*-like spore chains with extensive hairy ornamentation from Gruta da Terra Mole (Terceira Island, Azores); **(G)** Aggregates of coccoid cells with smooth surface and spherical cells arranged in chains resembling beads-on-a-string (arrow) from Bird Park Cave (Hawai'i, U.S.A.); **(H)** Chain of *Streptomyces*-like arthrospores from Honda del Bejenado Cave (La Palma Island, Spain); **(I)** Spores with spiny ornamentation from Helmcken Falls Cave, (British Columbia, Canada); **(J)** Spiral spore chains of *Streptomyces* and a coccoid cell with obtuse protuberances (arrow) from Falda de La Horqueta Cave (La Palma Island, Spain); **(K)** Large spheres with lumpy surface or protuberances connected by a network of filaments or appendages from Gruta dos Montanheiros (Pico Island, Azores); **(L)** CaCO_3_ spheres coated with a filamentous network from the Tapa Section of the Kipuka Kanohina Cave Preserve (Hawai'i, U.S.A.).

SEM images revealed the presence of possible Actinobacteria-like structures in most of the volcanic caves from all over the world showing a large variety of microbial morphologies and spore surface ornamentation (Figure 2). To confirm this observation, Sanger and pyrosequencing were performed. In general, these microbial mats were formed by a tangled mass of hyphae, spores, filamentous and coccoid cells (Figures 2A–C). Coccoid elements, with a diameter of about 0.5 μm, are frequently found in close heaps, intermingled with filamentous forms (Figures 2B,E). Most of these masses exhibited characteristic arthrospores of *Streptomyces* or close relatives with hairy (Figures 2C,F), smoothly (Figure 2H), spiny (Figure 2I) surface ornamentations. Spirals at the end of the aerial mycelium were also observed (Figure 2J). A notable feature of some of these bacteria is their filamentous growth with true branching, as depicted for instance in Figure 2H. Chains of coccoid cells resembling beads-on-a-string (Figure 2G) were found within both white and yellow mats. Some other microbial structures were difficult to associate to specific genera or species (Figures 2D,E,G,K,L). In addition, large spheres with lumpy surface connected by a network of hairy filaments and EPS (Figure 2K) or CaCO_3_ spheres (EDS microanalysis) coated with a filamentous network (Figure 2L) were occasionally observed in the colored microbial mats. Average sizes varied between 10 and 15 μm.

**Figure 3:**
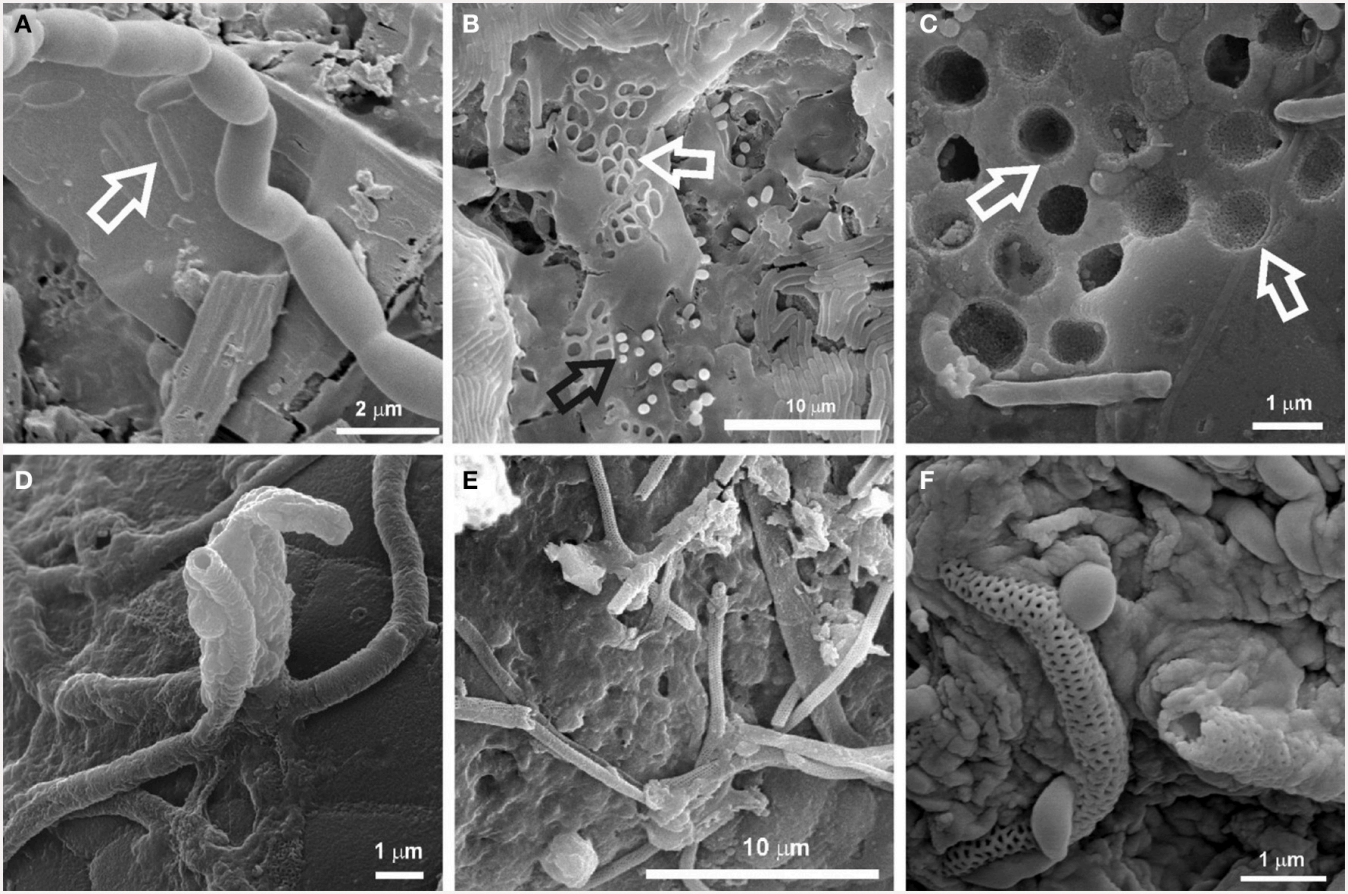
**SEM images of biosignatures found associated with microbial mats in Azorean, Canadian, Canarian, and Hawaiian volcanic caves. (A)** Cell-shaped etched pits on mineral grain (arrow) from Helmcken Falls Cave (British Columbia Canada); **(B)** Cell imprints (white arrow) and rods on EPS matrix from a white microbial mat in Gruta da Terra Mole (Terceira Island, Azores, Portugal); **(C)** Microborings produced by euendolithic cells on silicified mineral grains from Ana Heva cave in Easter Island, Chile (adapted from Miller et al., 2014); **(D)** Tubular mineralized sheaths embedded in EPS found on black deposits from Cueva del Llano de los Caños cave(La Palma Island, Spain). **(E)** Reticulated filaments found in white microbial mats in the Kula Kai Caverns of the Kipuka Kanohina Cave Preserve (Hawai'i, U.S.A.); **(F)** Close-up view of mineral encrusted filaments with reticulated ornamentation associated with white microbial mats on ooze-like deposits from Fuente de la Canaria cave (La Palma Island, Spain).

The microbial mats studied in this work were found to be involved in microbe-mineral interactions as revealed by SEM investigations (Figure 3). Cell-sized etch pits attributed to dissolution of the substrate under attached cells were noticed (Figures 3A–C). Microboring caused by euendolithic growth of coccoid cells was particularly evident on the silicified substrate, leaving imprints of their surface ornamentation on the mineral grains (Figure 3C). These microbial mats may also figure prominently in the deposition of minerals due to the presence of filaments, some of which are coated with minerals (Figures 3D–F). Among them, reticulated filaments similar to those reported by Melim et al. (2008) and Miller et al. (2012a) were found associated with the white microbial mats from the Kula Kai Caverns of the Kipuka Kanohina Cave Preserve (Hawai'i, U.S.A.) and Falda de La Horqueta cave, in La Palma Island, Spain (Figures 3E,F). All these features evidence microbe-mineral interactions and may represent mineralogical signatures of life. Both constructive and destructive mineral features in caves have been recognized as biosignatures valuable for the searching of traces of life on Earth and other planets (Banfield et al., 2001; McLoughlin et al., 2007; Hofmann et al., 2008; Northup et al., 2011).

The role of microorganisms in biomineralization and rock-weathering processes in caves has been discussed in recent years (Cuezva et al., 2012; Porca et al., 2012; Saiz-Jimenez, 2012). Both processes involve destruction and construction of mineral structures. Destructive processes include dissolution, etching or pitting, whereas constructive processes comprise precipitation of secondary minerals, such as calcite, struvite, witherite, and birnessite. In terms of weathering of minerals, the major processes promoted by microorganisms are biochemical and biophysical mechanisms of etching, dissolution and boring occurring via mechanical attachment and secretion of exoenzymes or organic acids (Lee et al., 2012). Extensively etched mineral grains such as calcite and Mg-silicate minerals were found associated with actinobacterial morphologies on coralloid-type speleothems from the Ana Heva volcanic lava tube cave in Chile (Miller et al., 2014). In many cases, it is difficult to determine the exact mechanism by which microorganisms induce mineral dissolution, but the pitting of underlying mineral grains, as shown in Figure 3, illustrates that it does occur.

On the other hand, microorganisms may directly precipitate minerals as part of their metabolic activity, and they can also indirectly impact mineral formation by altering the chemical microenvironment such as pH or redox conditions or providing nucleation sites for precipitation through the production of organic polymers (Benzerara et al., 2011). Numerous biogenic minerals have been reported in subterranean environments (Sanchez-Moral et al., 2003, 2004; Spilde et al., 2005; De los Ríos et al., 2011; Miller et al., 2012b, 2014), and some of them have been associated with actinobacterial communities. Laiz et al. (2003) found that 61% of the Actinobacteria isolated from Altamira Cave (Spain) produced mineral crystals on culture media. In general, culture and field sample biominerals were composed of calcite, aragonite, Mg-calcite or vaterite. Groth et al. (2001) also tested the ability of cave-dwelling bacteria from Grotta dei Cervi (Italy) for producing mineral crystals. These authors reported extensive mineral production among Actinobacteria, which induced the precipitation of calcite (e.g., *Brachybacterium* sp.) or vaterite (e.g., *Rhodococcus* sp.). Needle-fiber mats were also related to biomineralization processes by actinomycetes (Cañaveras et al., 1999, 2006). Struvite was formed by Actinobacteria isolated from tuff in Roman catacombs (Sanchez-Moral et al., 2003), and witherite, a naturally occurring barium carbonate, was produced by species of the genera *Agromyces* and *Streptomyces* isolated from tuff (Sanchez-Moral et al., 2004). Calcium carbonate spheres closely related to dense networks of interwoven filaments were observed within the colored microbial mats from Azorean, Canarian and Hawaiian volcanic caves (Figure 2L). Similar spherical particles were previously reported by Cuezva et al. (2012) and Diaz-Herraiz et al. (2013), who proposed vaterite as their mineralogical phase. According to Cuezva et al. (2012) the gray colonies found on Altamira cave walls, dominated by Actinobacteria, were able to bioinduce CaCO_3_ precipitation.

### Actinobacterial diversity found in New Mexico (USA), Hawai'i (USA), and Azores islands (Portugal)

**Figure 4:**
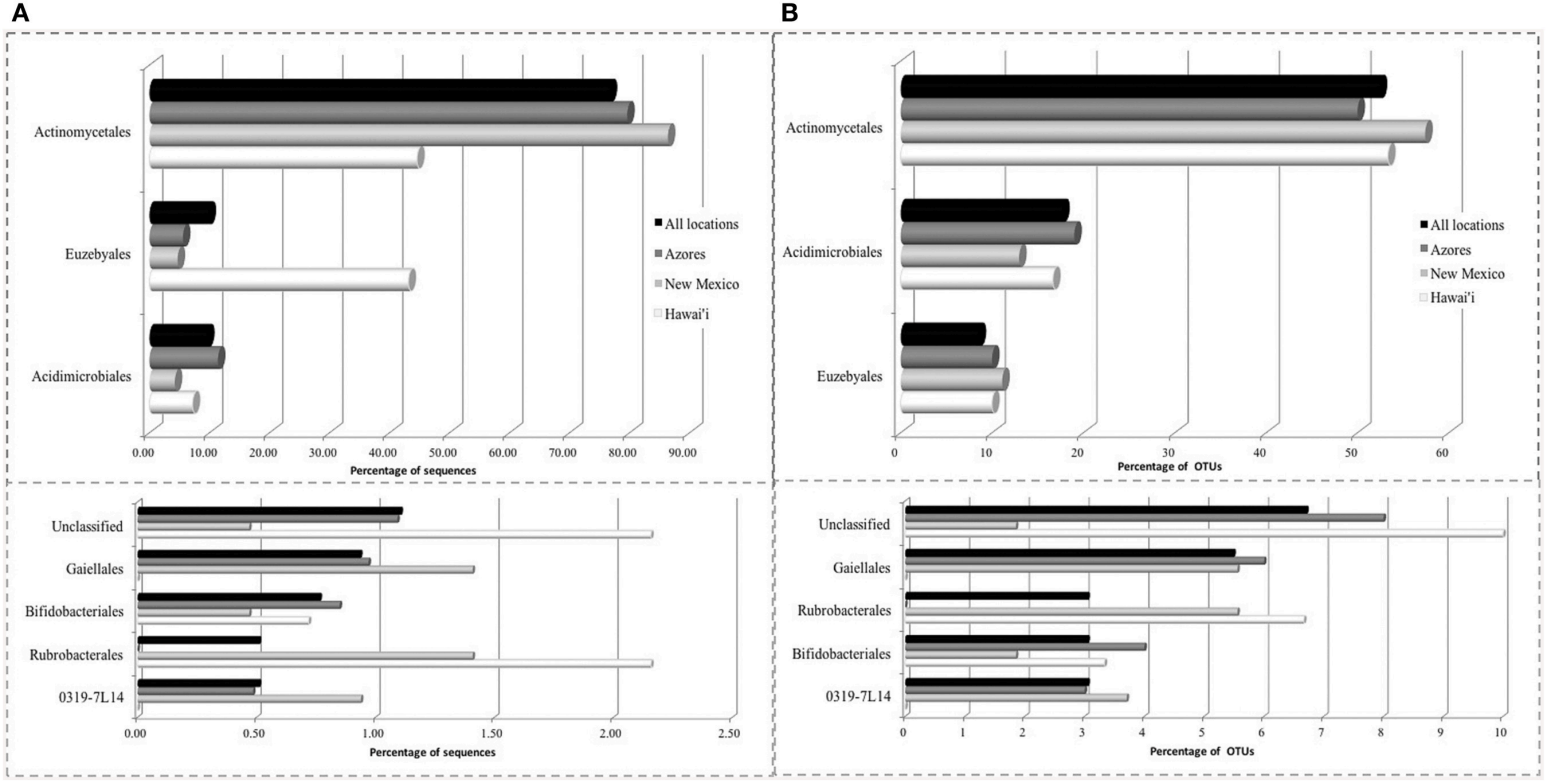
**Order-Level delineation of the 16S rRNA gene sequences (A) and OTUs (B) in Azores, New Mexico and Hawai'i lava caves**.

The SEM study revealed notable microbial diversity. In order to confirm the presence of Actinobacteria in these volcanic caves and further investigate their diversity, three geographically distinct locations, New Mexico (USA), Hawai'i (USA) and Azores islands (Portugal), were chosen for clone library analysis. A total of 1176 Actinobacteria sequences generated by clone libraries were determined to be of high quality and used in this analysis (Supplemental Table 1). These sequences clustered into 164 OTUs across all locations, belonging to seven orders. *Actinomycetales* (sequences = 76.7%, OTUs = 52.4%), *Euzebyales* (9.9%, 8.5%) and *Acidimicrobiales* (9.6%, 17.7%) represented the majority of the OTUs (Figures 4A,B, upper panel). *Bifidobacteriales* (0.8%, 3.0%)*, Gaiellales* (0.9%, 5.5%)*, Rubrobacterales* (0.5%, 3.0%), and candidate *0319-7L1* (0.5%, 3.0%) represented less than 1% of the sequences (Figures 4A,B, lower panel). Sequences that could not be assigned to taxonomic affiliations were labeled as “unclassified” (1.1%, 6.7%). Singletons and doubletons were the most common OTU type over all (116 singletons, 23 doubletons). Of the doubletons, 14 had two sequences from the same cave, and 20 had sequences from the same location.

Five of the OTUs (3.05%) represented 74.1% of the total number of sequences found. The most predominant OTU (OTU 025) belonged to the *Pseudonocardiaceae* family, with 593 sequences (50.4%) in 59 of the 82 samples. The second most common OTU (OTU 089), also a *Pseudonocardiaceae*, had 98 sequences (8.3%) in 29 samples, but was not found in Hawai'i. *Pseudonocardiaceae* was the most commonly found sequence and OTU in each location. This finding is consistent with other cave studies, which found *Pseudonocardiaceae* to comprise 52% of actinobacterial sequences in Carlsbad Cavern (Barton et al., 2007), 30–50% in three Slovenian limestone caves (Porca et al., 2012), and the most abundant OTU in a limestone cave in China (Wu et al., 2015).

**Figure 5:**
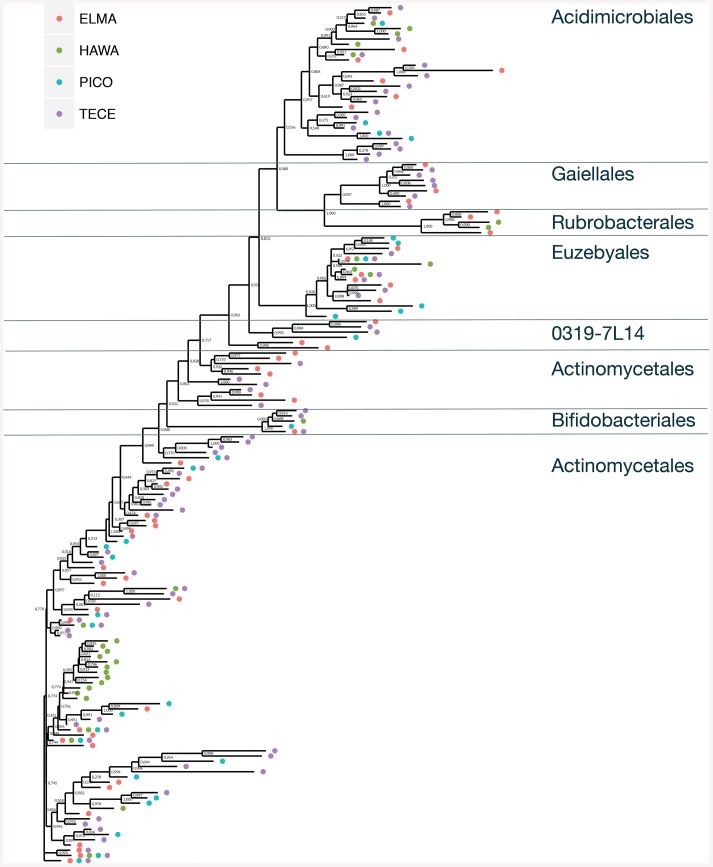
**Unrooted approximate maximum likelihood tree showing the relationship and occurrences of Actinobacteria OTUs across all four sample locations**. Bootstrap values are indicated.

OTUs belonging to the orders *Actinomycetales, Euzebyales, Acidomicrobiales*, and *Bifidobacteriales* were shared by at least two of the three locations under study (Figure 5, Supplemental Figure 1A). These ubiquitous OTUs may represent a core subsurface microbiota, a hypothesis that we will test in the future with more extensive sequencing. Furthermore, caves are not homogeneous habitats: they are characterized by zonal environments according to the distance to entrances (Poulson and White, 1969; Howarth, 1983, 1993), passage geometry, and microenvironments, which result from several types of reactions, including microbial processes that often involve redox reactions (Barton and Northup, 2007).

The number of shared OTUs in the three locations was relatively low; three out of five belonged to *Pseudonocardiaceae* and two were *Euzebyales* (Supplemental Figure 1A). Azores and New Mexico shared six other OTUs, four *Pseudonocardiaceae*, one *Euzebyales* and one *Bifidobacteriales*. Both archipelagos shared two *Acidomicrobiales*, one *Pseudonocardiaceae* and one unclassified OTU. Chao 1 estimator suggests that even though a more comprehensive sampling is required to provide a more complete assessment of these microbial communities, our sampling effort was probably enough to describe the cosmopolitan OTUs (Supplemental Figure 1B).

None of the sequences recovered were classified as *Streptomyces*, which was odd, given that *Streptomyces* are present in almost every other environment studied (i.e., soil, marine, etc., Schrempf, 2006), and were found in cultured isolates from the Azores (Riquelme and Dapkevicius personal communication). We believe this anomaly is due to primer bias. Farris and Olson (2007) showed that many Actinobacteria were not amplified in PCR despite being 100% identical to the universal primers used. While this does not conclusively establish that our sequencing missed *Streptomyces* that are present, it is cause for concern. Future sequencing efforts will utilize Actinobacteria-specific primers to test our hypothesis that *Streptomyces* are being missed and to better characterize the diversity of the Actinobacteria in caves.

**Figure 6:**
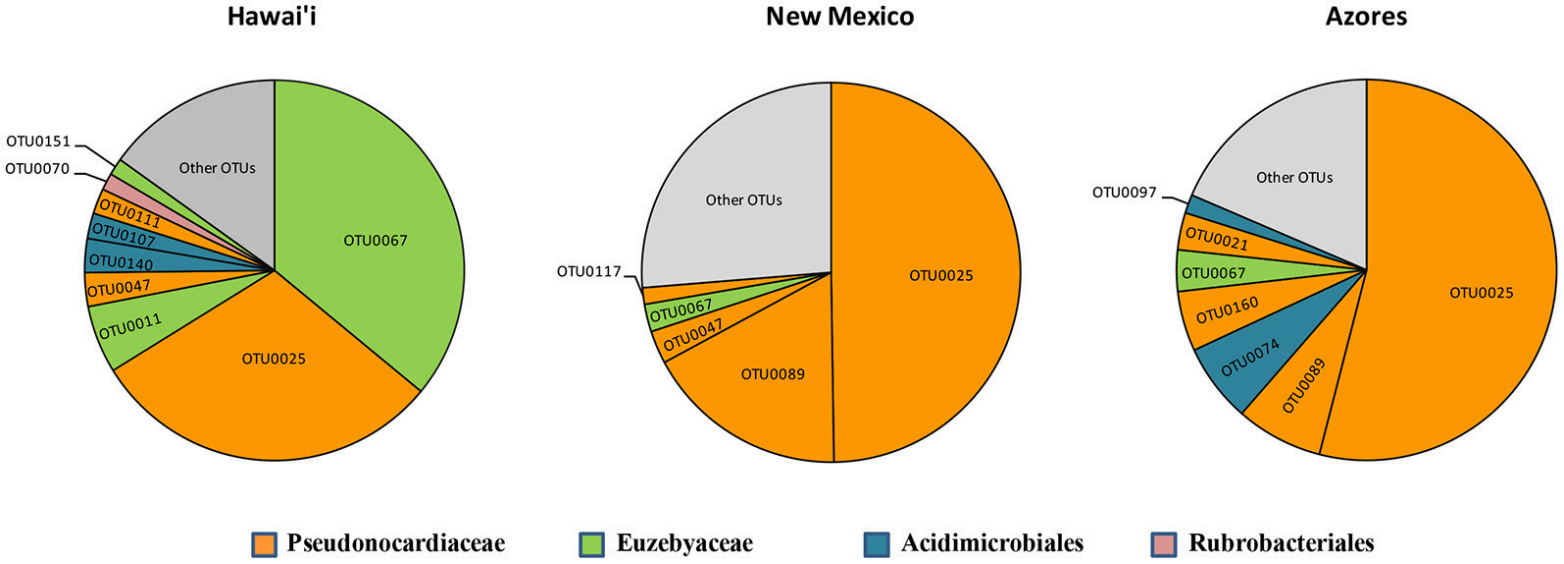
**Most abundant OTUs for Hawai'i, New Mexico, and Azores**. OTUs were clustered at 97% similarity and represent more than 1% of the total sequences recovered in each geographical location.

*Euzebyales* emerged as the second most abundant order (number of sequences) in New Mexico and Hawai'i; however, *Acidomicrobiales* had the second most OTUs in New Mexico and Hawai'i, and was second for both sequences and OTUs in the Azores (Figure 6). *Euzebyales* was recently described and has two known genera (Kurahashi et al., 2010), and highly similar sequences have been identified from numerous environments (sea cucumbers, saline soils and caves) suggesting this order may be widespread in numerous habitats (Cuezva et al., 2012; Ludwig et al., 2012; Ma and Gong, 2013; Velikonja et al., 2014). The *Acidimicrobiales* order was described by Stackebrandt et al. (1997) and comprises members that are obligate acidophiles, oxidize ferrous iron or reduce ferric iron. It has already been described in caves (Macalady et al., 2007; Ortiz et al., 2013; De Mandal et al., 2014), other volcanic environments (Cockell et al., 2013) and Fe-rich environments (Sánchez-Andrea et al., 2011; Grasby et al., 2013).

### Evaluation of diversity coverage and richness of the clone libraries

The coverage average estimated for the different locations ranged from 78 to 86%. Due to some variation in sampling effort in each case, a re-sampling analysis was performed, randomly selecting the smallest number of sequences across the different groups (139), 1000 times per each sample, to standardize the values. Diversity indices and estimators are summarized in Table 1A. Non-parametric Shannon and Shannon suggested more diverse communities within New Mexico caves compared to Hawai'i and Azores. Simpson diversity indices suggest the highest diversity values for Hawai'i. All indices agree with the less diverse communities being in Azores. The Shannon index gives more weight to the rare species and Simpson to the dominant ones. Considering the Simpson indexes of the three locations, the community composition in Azores caves would include more cosmopolitan species with high abundance and Hawai'i caves would be composed of phylotypes with narrower distribution. In islands, population size and genetic diversity tend to be limited due to the smaller extension of the habitats. Comparable taxa–area relationships (Bell et al., 2005) and distance–decay relationships for microbes and larger organisms were found to be significant although with variations in the rates of the processes (reviewed by Green and Bohannan, 2006; Soininen, 2012). However, we found differences between the diversity indices for Azores and Hawai'i, which could be related to differences in island size, isolation and age of lava flows. We should be aware that the amount of data available is still small and that further studies may still reveal different trends.

**Table 1A:** **Summary of the observed richness, diversity indices, coverage, and Chao 1 richness estimator at 97% similarity level at the three locations under study**.

	**Azores**	**New Mexico**	**Hawai'i**
Richness	29.19	38.52	30
Shannon	2.01 (1.73–2.29)	2.16 (1.86–2.46)	2.13 (1.87–2.39)
Npshannon	2.35	2.69	2.48
Simpson	0.31 (0.22–0.39)	0.28 (0.20–0.35)	0.22 (0.17–0.27)
Invsimpson	3.31 (2.60–4.56)	3.62 (2.86–4.93)	4.49 (3.68–5.78)
Chao	97.79 (51.97–240.21)	158.98 (82.10–374.89)	100 (51.51–257.75)
coverage	0.86	0.78	0.85

### Phylogenetic analysis

**Figure 7:**
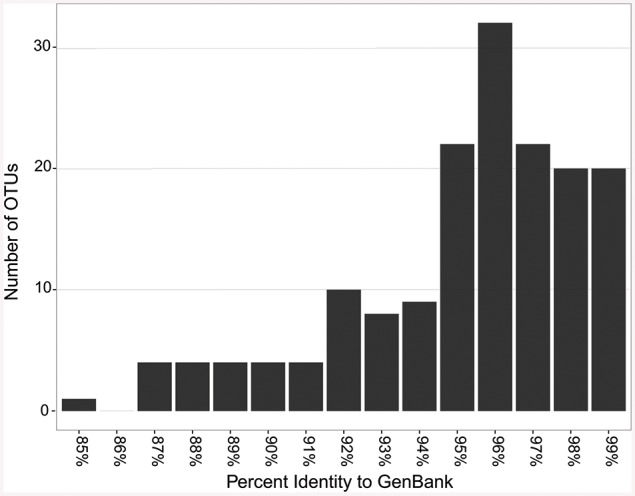
**Sequence identity based on BLAST comparisons to GenBank**.

When the representative sequences from each OTU were compared to known sequences in GenBank, 17 out of 164 OTUs (10%) shared ≤90% identity with known sequences in GenBank (Figure 7). Fifty two percent of the OTUs shared between 91 and 96% identity and 38% shared over 97% identity with known sequences. The most novel OTUs were mostly singletons, and were classified as *Pseudonocardiaceae* (four OTUs), *Rubrobacteraceae* (one OTU), *Bifidobacteriales* (five OTUs), and unclassified (seven OTUs). They were found in all four locations, however, more of the OTUs were found in the Azorean islands (13 out of 100) than in Hawai'i (2 out of 30) or New Mexico (3 out of 54). Physical isolation is an important driver of microbial evolution (Papke and Ward, 2004); thus, island isolation would promote unique evolutionary forces that result in the development of a novel genetic reservoir. However, in our results we did not observe significant differences between continental and island territories according to genetic novelty.

An approximate maximum likelihood tree shows the relationship between the sequences and occurrence of OTUs (Figure 5). For this analysis Pico and Terceira were considered separate locations. *Gaiellaceae*-like sequences were found in New Mexico and the Azores, but not Hawai'i. All but one of the sequences were singletons. *Gaiellaceae*, another recently described family, was originally found in a water borehole, and sequences from this family have subsequently been found in soil, volcanic soil, thermal springs and marine ascidians (Albuquerque et al., 2011; Kim et al., 2014; Rozanov et al., 2014; Steinert et al., 2015). *Rubrobacterales* occurred in the New Mexico and Hawai'i samples. The order *Actinomycetales* has many polytomies with most of them occurring in the samples from Hawai'i, Pico, and Terceira. These samples are either unresolved parts of the tree due to missing data or represent rapid speciation in the *Actinomycetales*. Representatives of *Euzebyales* were found in all four locations (Figure 5). The different clades suggest there is significant diversity within the sequences found.

While we acknowledge the limitation of our study to capture the full range of diversity in these sites, the high number of singletons found in this study suggests that there are Actinobacteria belonging to the rare biosphere in caves. The rare biosphere has been shown to influence both alpha and beta diversity, exhibiting unique geographic patterns (Lynch and Neufeld, 2015).

With over two thirds of our OTUs being singletons and most of the doubletons from one location, there is evidence to suggest endemism in cave Actinobacteria. Endemism in caves has been documented for obligate cave fauna in the United States and the Azores (Culver et al., 2003; Reboleira et al., 2012). Furthermore, studies of Actinobacteria in other environments have been shown to display endemism (Wawrik et al., 2007; Valverde et al., 2012). The combination of rare and endemic Actinobacteria, together with their abundance in caves, support the idea that caves are a good location to further test hypotheses regarding bacterial biogeography as well as to look for novel actinobacterial metabolites. Rigorous testing will require that future studies be conducted with next generation sequencing to comprehensively sample the diversity present in these habitats.

### 16S rRNA gene amplicon library preparation, sequencing, bioinformatics, and OTU-based analysis in Azorean Volcanic Caves

The observed structure of the microbial communities in volcanic caves in the three locations is consistent with bacterial communities composed of consortia of few cosmopolitan members and a high number of low abundant phylotypes. To test whether this structure could be biased by the fact of having a limited number of sequences, a pyrosequencing approach was performed with the same sample points considered for clone libraries in Azores.

Actinobacterial sequences amplified using the universal primers were identified and after quality control and filtering of the crude pyrotags, 19,476 sequences with good quality were retained, consisting of 906 unique sequences. The average sequence length was 247.5 bp (range 233–275; median 247.1; sd 4.1). After clustering, a total of 382 OTUs were obtained.

**Figure 8:**
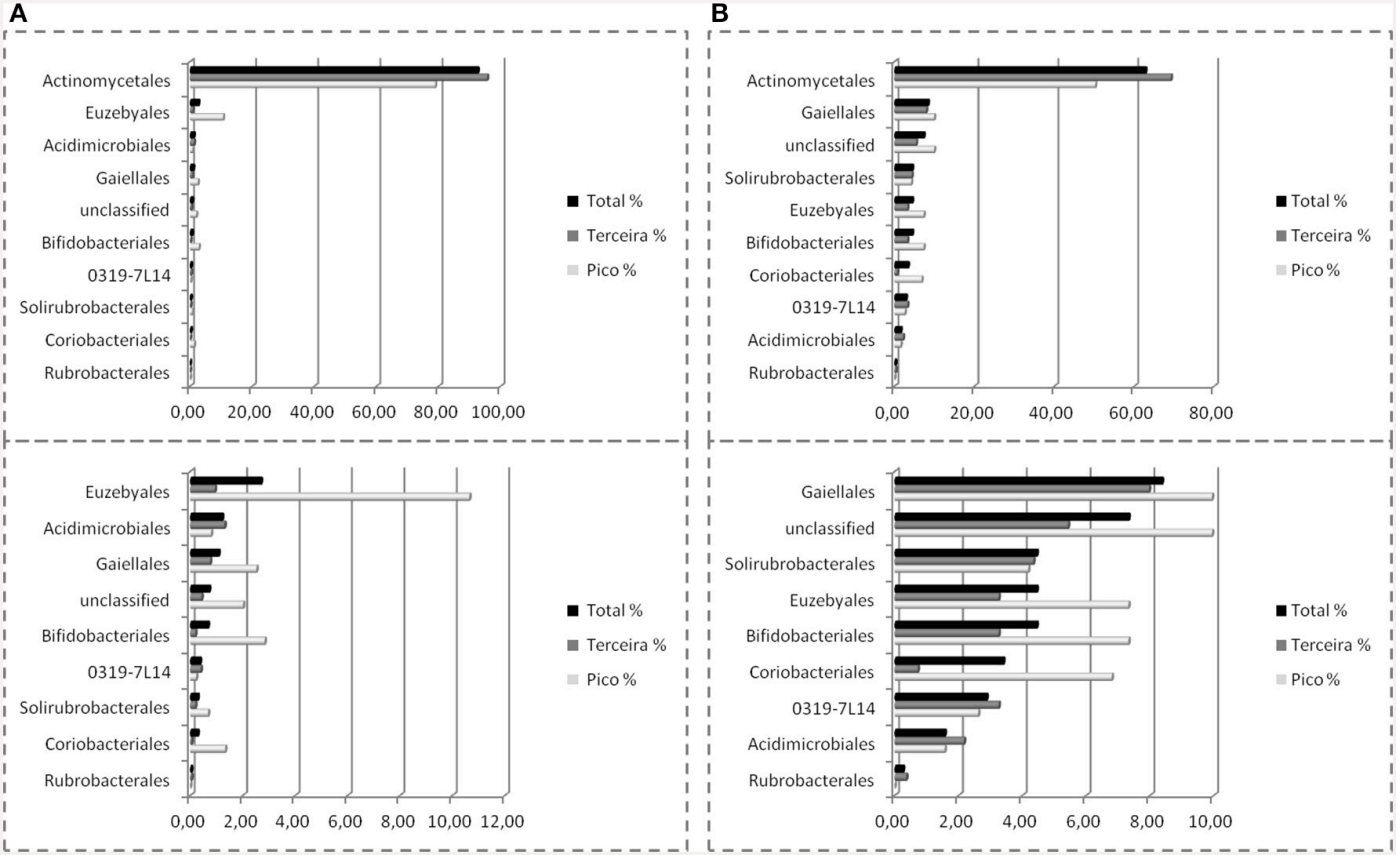
**Order-Level delineation of the 16S rRNA gene sequences (A) and OTUs (B) in Azorean volcanic caves obtained by amplicon library**.

Nine orders were found in Azorean caves with pyrosequencing, the seven previously found, i.e., candidate *0319-7L1* (sequences = 0.4%, OTU = 2.9%), *Acidimicrobiales* (1.2%, 1.6%), *Actinomycetales* (92.6%, 62.8%), *Bifidobacteriales* (0.7%, 4.5%), *Euzebyales* (2.7%, 4.5%), *Gaiellales* (1.1%, 8.4%)*, Rubrobacterales* (0.04%, 0.3%), plus *Coriobacteriales* (0.3%, 3.4%), and *Solirubrobacterales* (0.3%, 4.5%) (Figure 8). While *Rubrobacterales* was found in the clone libraries, it was only found in New Mexico and Hawai'i (Figure 5). Amplicon sequencing revealed this order to be present in the Azores as well, highlighting the importance of pyrosequencing to capture the full range of diversity in these samples. *Actinomycetales* and *Gaiellales* orders showed an increase in the percentage of sequences and OTUs recovered; *Bifidobacteriales* had a higher percentage of OTUs. All other orders displayed lower percentages both for sequences and OTUs. Unclassified sequences represented 0.7 and 7.3%, respectively.

The amplicon libraries approach showed a more complete picture of the subterranean diversity in Azorean volcanic caves. *Rubrobacterales* comprised a group of novel OTUs, with all sequences sharing no more than 92% similarity with known sequences in GenBank, as well as *Solirubrobacterales*, with all of the sequences ranging between 90 and 95% similarity (Stackebrandt et al., 1997; Reddy and Garcia-Pichel, 2009). *Rubrobacterales* was first described in cave environments in Niu Cave (Zhou et al., 2007), and were also recovered from speleothems in Kartchner Caverns. This order includes members with heat, cold, dryness and high radiation resistance, found in high number in biodeteriorated monuments (Gurtner et al., 2000; Jurado et al., 2012) and volcanic environments (Cockell et al., 2013). *Solirubrobacterales* have also been described in caves (Paterson, 2012; De Mandal et al., 2014) and in other volcanic environments (Gomez-Alvarez et al., 2007; Cockell et al., 2013). *Coriobacteriales* (Stackebrandt et al., 1997; Gupta et al., 2013) showed a high percentage of sequences, 89.1%, with more than 97% similarity. This order was previously described in cave habitats in speleothem formations in Kartchner Caverns (Ortiz et al., 2013), and in Lower Kane cave (Paterson, 2012).

### Evaluation of diversity coverage and richness of the amplicon libraries

Sampling completeness assessed by Good's coverage estimator for each data set returned values above 98% (Table 1B). Diversity indices revealed a higher diversity at Pico Island compared to Terceira Island as well as chao richness estimator (Table 1B).

**Table 1B:** **Summary of the characteristics of the pyrosequencing data**.

	**Azores**	**Pico**	**Terceira**
Richness	382	191	141.57
Shannon	1.99 (1.96–2.02)	2.67 (2.60–2.75)	1.68 (1.61–1.75)
Npshannon	2.04	2.76	1.79
Simpson	0.40 (0.40–0.41)	0.24 (0.23–0.26)	0.45 (0.43–0.46)
Invsimpson	2.48 (2.43–2.53)	4.11 (3.88–4.37)	2.25 (2.15–2.35)
Chao	529.70 (477.26–611.02)	262.19 (229.43–322.89)	256.86 (203.65–355.78)
Coverage	0.99	0.98	0.98

*I.e., observed richness, diversity indices, coverage and Chao 1 richness estimator at 97% similarity level at the two islands from the Azorean archipelago under study*.

The dominance of the *Pseudonocardiaceae* family compared to any other member of the microbial community is remarkable, in accordance with results from both clone and amplicon libraries. *Pseudonocardiaceae* encompases a wide array of rare *Actinomycetes*, many of which can produce secondary metabolites (Tiwari and Gupta, 2013). While we acknowledge that this finding may be in part the result of primer bias, the prevalence of this family is not uncommon in caves (Barton et al., 2007; Porca et al., 2012; Wu et al., 2015). Little is known of role these bacteria play in most ecosystems, however the family encompases a wide variety of metabolic pathways and physiologies (Huang and Goodfellow, 2011). Most of our sequences were unable to be classified at the genus level, leaving some doubt as to the true role of this group of bacteria in volcanic caves. However, the ubiquity of this family in cave studies emphasizes the need for further molecular studies with improved primers to capture Actinobacteria diversity and cultivation of members of this family found in subterranean bacterial biofilms. An examination of the communities *in situ* combined with metatranscriptome analysis would shed light on the question of this group's role in volcanic cave ecosystems.

## Conclusions

Our collective attempt to better understand actinobacterial diversity and functions in volcanic caves led us to observe patterns of diversity and novelness through a range of data obtained from 454 pyrosequencing to cloning. To date, within the realm of actinobacterial community study, our work is one of the largest sampling efforts in volcanic caves from different parts of the world including Spain, Portugal, USA and Canada. The sequencing effort, both in clone and amplicon libraries, represents one of the most comprehensive studies of Actinobacteria in volcanic caves around the world. The clone libraries illustrate the novelness and phylogenetic relationship of Actinobacteria in volcanic caves from three geographically distant locations. The amplicon libraries of the Azorean sequences gave a more in-depth view of the Actinobacteria communities and revealed more diversity than has previously been described. Both methods showed large numbers of newly described orders, and a dominance of *Actinomycetales*. Together they provide an outline of the community structure of Actinobacteria in caves, and highlight the importance of caves as a source of rare and novel Actinobacteria.

Through scanning electron microscopy examinations, we learned about bacterial morphology, their relationships and possible contribution of the Actinobacteria to cave environment. The identification of Ca-rich elements coated within some of the filamentous networks in the colored microbial mats suggests a possible role of Actinobacteria in calcium deposition. Both constructive and destructive mineral features, such as biominerals, cell imprints, microboring and mineralized filaments were some of the biosignatures found associated with samples studied herein. We can thus consider that volcanic caves on Earth are plausible repositories of terrestrial biosignatures where we can look for evidence of early life.

Beyond contributing to understanding cave microbial ecology, community and microbial roles and related function in such extreme subsurface habitats, our study hopes to initiate more study in such an interesting and understudied frontier of the Earth, where unique compounds could be isolated and used as important sources of industrial processes.

### Conflict of interest statement

The authors declare that the research was conducted in the absence of any commercial or financial relationships that could be construed as a potential conflict of interest.

## Footnotes

^1^www.ncbi.nlm.nih.gov/BLAST/.

^2^www.mbio.ncsu.edu/BioEdit/bioedit.html.
