# Supplementary material for: Actinobacterial Diversity in Volcanic Caves and Associated Geomicrobiological Interactions
Source: Front Microbiol. 2015 Dec 9;6:1342. doi: 10.3389/fmicb.2015.01342 (PMC4673402; doi:10.3389/fmicb.2015.01342)
Supplement: Supplementary file 1 [file DataSheet1.DOCX]

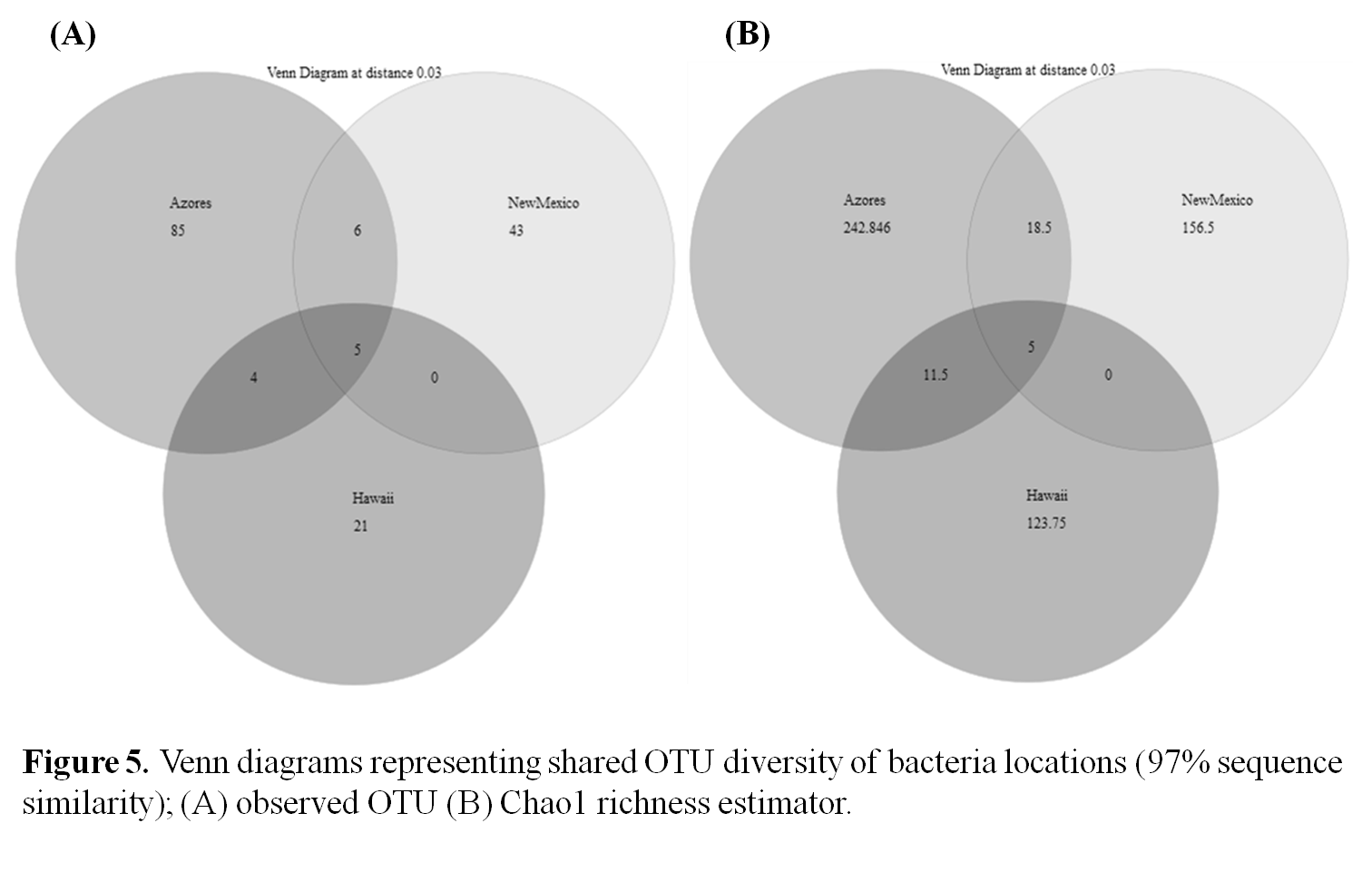


**Supplemental Figure 1**. Venn diagrams representing shared OTU diversity of bacteria locations (97% sequence similarity). (A) Observed OUT; (B) Chao1 richness estimator.

**Supplemental Table 1.** Location and number of samples and sequences from each cave.

|  |  | **Clone libraries** | | **Amplicon libraries** | |
| --- | --- | --- | --- | --- | --- |
| **Location** | **Cave** | **Number of samples** | **Number of sequences** | **Number of samples** | **Number of sequences** |
| Hawai’i | Bird Park | 3 | 61 | - | - |
|  | Epperson’s | 1 | 19 | - | - |
|  | Kaumana | 3 | 30 | - | - |
|  | Maelstrom | 1 | 2 | - | - |
|  | Thurston | 1 | 2 | - | - |
|  | Kula Kai caverns | 2 | 25 | - | - |
| New Mexico | Cave 12 | 2 | 19 | - | - |
|  | Cave 255 | 1 | 56 | - | - |
|  | Cave 266 | 3 | 17 | - | - |
|  | Cave 261 | 3 | 44 | - | - |
|  | Cave 315 | 3 | 77 | - | - |
| Pico | Furna do Lemos | 3 | 62 | 2 | 395 |
|  | Gruta dos Montanheiros | 1 | 4 | 2 | 521 |
|  | Gruta da Ribeira do Fundo | 4 | 63 | 2 | 1043 |
|  | Gruta das Torre | 4 | 110 | 4 | 1562 |
| Terceira | Algar do Carvão | 3 | 41 | 2 | - |
|  | Gruta das Agulhas | 3 | 25 | 2 | 1137 |
|  | Gruta da Achada | 3 | 52 | 2 | 1600 |
|  | Gruta dos Buracos | 3 | 39 | 2 | 3477 |
|  | Gruta dos Balcões | 3 | 43 | 2 | 1601 |
|  | Gruta da Branca Opala | 4 | 107 | 2 | 1846 |
|  | Gruta da Madre de Deus | 3 | 101 | 2 | 3800 |
|  | Gruta do Natal | 3 | 23 | 2 | 788 |
|  | Gruta da Terra Mole | 4 | 32 | 2 | 985 |
|  | Gruta dos Principiantes | 4 | 66 | 2 | 103 |
|  | Gruta da Malha | 5 | 56 | 2 | 618 |

|  |  |  |  |
| --- | --- | --- | --- |
|  |  |  |  |
|  |  |  |  |

**Supplemental Table 2.** GenBank Accession Numbers.

| Location | Accession Numbers |
| --- | --- |
| Hawai’i ^a^ | HM063012, HM063014-HM063020, HM063025, HM063027, HM444834, HM444836, HM444862, HM444887, HM444891, HM444892, HM444907, HM444912, HM444917, HM444919, HM444931, HM444932, HM444938, HM444947, M444948, HM444956, HM445516-HM445518, HM445523, HM445540, HM445549, HM445551, HM445559-HM445561, HM445565, KC569805, KC569806, KC569822, KC569827, KC569846, KC569875, KC569892, KC569894, KT167192, KT167193 |
| New Mexico | KC331621-KC331653, KC331655-KC331692, KC331694-KC331743, KC331745-KC331751, KC331753-KC331838 |
| Azores (Terceira) ^a^ | HM444993, HM444996, HM444998, HM445002, HM445004, HM445009, HM445017-HM445019, HM445029, HM445047, HM445082, HM445085, HM445102, HM445106, HM445113, HM445132, HM445148, HM445185, HM445192, HM445223, HM445238, HM445249, HM445251, HM445254, HM445255, HM445265, HM445268, HM445285, HM445326, HM445368, HM445393, HM445436, HM445437, HM445445, HM445486, JF265974, JF265979, JF265985, JF265988, JF265999, JF266010, JF266021, JF266027, JF266036, JF266037, JF266042, JF266065, JF266068, JF266077, JF266079, JF266084, JF266085, JF266093, JF266100, JF796752, JF796758, JF796763, JF796770, JF796771, JF796776, JF796777, JF796781, JF796798, JN592625, JN592629, JN592643, JN592647, JN592648, JN592652, JN592655, JN592660, JN592670, JN592678, JN592689, JN592701, JN592702, JN592709, JN600569, JN600572, JN600586, JN600593, JN600597, JN606986,, JN606990, JN606992, JN606998, JN607005, JN607006, JN607010, JN607015, JN607016, JN607017, JN607024, JN607028, JN607030, JN607033, JN607037, JN607041, JN607044, JN607045, JN607051, JN607052, JN607066, JN607069, JN607080, JN615637, JN615642, JN615645, JN615647, JN615657, JN615666, JN615668, JN615673, JN615686, JN615690, JN615700, JN615712, JN615726, JN615737, JN615745, JN615750, JN615751, JN615780, JN615793, JN615802, JN615805, JN615814, JN615818, JN615825, JN615828, JN615830, JN615831, JN615837, JN615841, JN615844, JN615851, JN615854, JN615855, JN615858, JN615859, JN615861, JN615863, JN615865, JN615867, JN615870, JN615873, JN615878, JN615880, JN615881, JN615888, JN615889, JN615891, JN615892, JN615896, JN615897, JN615899, JN615900, JN615901, JN615902, JN615906, JN615907, JN615910, JN615913, JN615919, JN615923, JN615925, JN615928, JN615933, JN615946, JN615951, JN615954, JN615959, JN615960, JN615962, JN615963, JN615965, JN615966, JN615971, JN615973, JN615977, JN615979, JN615980, JN615981, JN615982, JN615985, JN615988, JN615989, JN615994, JN615995, JN615996, JN616002, JN616003, JN616005, JN616009, JN616011, JN616012, JN616016, JN616018, JN616019, JN616020-JN616022, JN616028, JN616030-JN616032, JN616034, JN616035, JN616040, JN616041, JN616044, JN616046, JN616047, JN616056-JN616060, JN616063, JN616064, JN616068, JN616073, JN616077, JN616079-JN616083, JN616085, JN616087, JN616089, JN616091, JN616095, JN616096, JN616102, JN616106-JN616108, JN616110, JN616115, JN616116, JN616120, JN616124, JN616133-JN616135, JN616140, JN616142, JN616143, JN616146, JN616147, JN616149, JN616156, JN616157, JN616160, JN616172, JN616178, JN616179, JN616181-JN616183, JN616185, JN616188, JN616191, JN616194, JN616205, JN616209, JN616211, JN616215, JN616219, JN616222, JN616223, JN616224, JN616227, JN616231-JN616233, JN616237-JN616239, JN643002, JN643009, JN643019, JN672040, JN672045-JN672047, JN672049, JN672050, JN672053, JN672061, JN672065, JN672066, JN672068, JN672075-JN672077, JN672080, JN672082, JN672088, JN672090, JN672091, JN672095, JN672096, JN672098, JN672103, JN672105, JN672108, JN672109, JN672110-JN672113, JN672116, JN672118-JN672120, JN672122, JN672125, JN672126, JN672142, JN672148, JN672160, JN672184, JN672197, JN672202, JN672205, JN672207, JN672208, JN672216-JN672218, JN672220, JN672222, JN672223, JN672224, JN672225, JN672227-JN672230, JN672233, JN672236, JN672237, JN672239, JN672241, JN672242, JN672251, JN672254, JN672257, JN672258, JN672263, JN672265, JN672268, JN672280, JN672283, JN672292, JN672293, JN672296, JN672311, JN672320, JN672332, JN672339, JN672344, JN672358, JN672378, JN672400, JN672416, JN672420, JN672486, JN672520, JN672532, JN672543, JN672547, JN701036, JN701037, JN701041, JN701043-JN701046, JN701051, JN701056, JN701058, JN701060, JN701061, JN701064, JN701065, JN701067, JN701072, JN701075, JN701080, JN701084, JN701093, JN701094, JN701096, JN701099, JN701104-JN701109, JN701112, JN701114, JN701116-JN701120, JN701124, JN701125, JN701127, JN701131, JN701133, JN701135-JN701139, JN701141, JN701143, JN701144, JN701146-JN701149, JN701154, JN701156, JN701157, JN701159-JN701162, JN701164, JN701166, JN701168, JN701171, JN701173, JN701174, JN701176, JN701178, JN850065, JN850066, JN850067, JN850069, JN850070, JN850071, JN850073, JN850074, JN850076, JN850078-JN850081, JN850084, JN850086, JN850090, JN850091, JN850093, JN850095, JN850096, JN850099-JN850111, JN850114-JN850118, JN850120, JN850129, JN850150, JN850173, JN850176-JN850179, JN850182, JN850186, JN850188, JN850189, JN850191, JN850192, JN850194, JN850195, JN850198, JN850199, JN850202-JN850204, JN850207, JN850208, JN850210, JN850211, JN850213, JN850218, JN850223, JN850227, JN850233, JN850237, JN850241, JN850243, JN850244, JN850246, JN850250, JN850259, JN850261, JN850270, JN850271, JN850273, JN850280, JN850281, JN850284, JN850287, JN850288, JN850291, JN850293, JN850294, JN850295, JN850299, JN850302, JN850306, JN850307, JN850310, JN850311, JN850313-JN850315, JN850317, JN850319-JN850323, JN850325, JN850327-JN850330, JN850332-JN850338, JN850340-JN850342, JN850344-JN850352, JN850354-JN850358, JN850360-JN850362, JN850376, JN850419, JN850440, JN850442, JN850483, JN850517, JN850520 |
| Azores (Pico) | HM749656, HM749661, HM749663, HM749669, HM749671, HM749672, HM749674, HM749681, HM749683, HM749688, HM749689, HM749691, HM749697, HM749702, HM749703, HM749705, HM749707, HM749709, HM749710, HM749714, HM749723, HM749726, HM749734, HQ721104, HQ721105, HQ721111, HQ721114, HQ721115, HQ721120, HQ721127, HQ721137, HQ721147, HQ721148, HQ721152, HQ721154, HQ721155, HQ721161-HQ721163, HQ721168, HQ721169, HQ721170, HQ721172, JF265703, JF265714-JF265716, JF265718, JF265739, JF265742, JF265744, JF265825, JF265833, JF265843, JF265855, JF266105, JF266109, JF266113, JF266116, JF266120, JF266123, JF266124, JF266126, JF266127, JF266133, JF266146, JF266147, JF266161, JF266166, JF266168, JF266169, JF266171, JF266172, JF266174, JF266175, JF266179, JF266184, JF266187, JF266190, JF266196-JF266198, JF266200, JF266204 , JF266210, JF266229, JF266233, JF266236, JF266237, JF266238, JF266240, JF266249, JF266254, JF266255, JF266275, JF266280, JF266284, JF266287, JF266290, JF266292, JF266294, JF266298, JF266299, JF266301, JF266303, JF266309, JF266316, JF266323, JF266326, JF266329, JF266341, JF266343, JF266344, JF266346-JF266348, JF266350, JF266355, JF266360, JF266361, JF266365, JF266366, JF266369, JF266373-JF266381, JF266386, JF266388-JF266390, JF266393, JF266395, JF266396, JF266397, JF266400, JF266401-JF266403, JF266407, JF266410, JF266413, JF266416, JF266418, JF266419, JF266432, JF266448, JF266471, JF266483, JF266495, JF266523, JF266534, JF266540, JF266555, JN696293, JN696295, JN696301, JN696304, JN696305, JN696308, JN696311, JN698909, JN698912, JN698913, JN698915, JN698916, JN698917, JN698918, JN698919, JN698914, JN698920, JN698921-JN698925, JN698927-JN698935, JN698937-JN698942, JN698945, JN698947, JN801072, JN801073, JN801078, JN801084-JN801086, JN801088-JN801090,JN801095-JN801101, JN801103, JN801105, JN801107, JN801111, JN801113-JN801117, JN801122, JN801124,JN801126-JN801128, JN801130, JN801131, JN801133, JN801135, JN801136, JN802348-JN802350, JN802357, JN802363, JN802365, JN802367 |
| Azores 454-amplicons | KT230870-KT231775 |

a: The following accession numbers represent more than one sequence. HM444862 (2 sequences), HM444891 (41), HM444892 (2), HM444919 (8), HM444948 (13), HM445517 (25), HM445518 (2), HM445523 (2), HM445559 (3), HM445561 (2), JN850250 (2).
